# Integrated Effects of Co-Inoculation with Phosphate-Solubilizing Bacteria and N_2_-Fixing Bacteria on Microbial Population and Soil Amendment Under C Deficiency

**DOI:** 10.3390/ijerph16132442

**Published:** 2019-07-09

**Authors:** Zhikang Wang, Ziyun Chen, Xiangxiang Fu

**Affiliations:** Co-Innovation Center for Sustainable Forestry in Southern China, College of Forestry, Nanjing Forestry University, Nanjing 210037, China

**Keywords:** phosphate-solubilizing bacteria, N_2_-fixing bacteria, soil amendment, C deficiency

## Abstract

The inoculation of beneficial microorganisms to improve plant growth and soil properties is a promising strategy in the soil amendment. However, the effects of co-inoculation with phosphate-solubilizing bacteria (PSB) and N_2_-fixing bacteria (NFB) on the soil properties of typical C-deficient soil remain unclear. Based on a controlled experiment and a pot experiment, we examined the effects of PSB (M: *Bacillus megaterium* and F: *Pseudomonas fluorescens*), NFB (C: *Azotobacter chroococcum* and B: *Azospirillum brasilence*), and combined PSB and NFB treatments on C, N, P availability, and enzyme activities in sterilized soil, as well as the growth of *Cyclocarya Paliurus* seedlings grow in unsterilized soil. During a 60-day culture, prominent increases in soil inorganic N and available P contents were detected after bacteria additions. Three patterns were observed for different additions according to the dynamic bacterial growth. Synergistic effects between NFB and PSB were obvious, co-inoculations with NFB enhanced the accumulation of available P. However, decreases in soil available P and N were observed on the 60th day, which was induced by the decreases in bacterial quantities under C deficiency. Besides, co-inoculations with PSB and NFB resulted in greater performance in plant growth promotion. Aimed at amending soil with a C supply shortage, combined PSB and NFB treatments are more appropriate for practical fertilization at intervals of 30–45 days. The results demonstrate that co-inoculations could have synergistic interactions during culture and application, which may help with understanding the possible mechanism of soil amendment driven by microorganisms under C deficiency, thereby providing an alternative option for amending such soil.

## 1. Introduction

In Southern China, plantation areas are mostly assigned to poor sites with yellowish-brown clay soil in the subtropical mountainous areas, as part of the Grain for Green Project (GTGP). These regions are perceived to be infertile due to low levels of organic C and nutrients [1,2]. To improve the poor status of such soils, many studies are emphasizing efficient soil amendment strategies [1,3]. However, the most common strategy, chemical fertilization, has produced harmful effects in the soil and the environment [4]. Due to leaching and immobilization [5], few nutrients, such as N and P, in the soil are available for plant uptake even after long-term chemical fertilizer treatment [6,7,8]. These problems are now compelling researchers to find more sustainable and advanced techniques to remediate the soil [9,10]. Of the recommended strategies, the use of bio-fertilizer has proven to be an efficient and eco-friendly management practice in improving soil fertility and crop growth [11].

A bio-fertilizer is a substance containing living beneficial microorganisms that can colonize the rhizosphere and stimulate plant growth by increasing the supply of available nutrients to plants when applied to the soil [12]. Soil N and P are known to be two of the most essential nutrients for plant growth and development worldwide. As tested soils in South China are seriously lacking in available N and P, the fixation of N and solubilization of P driven by N_2_-fixing bacteria (NFB) and phosphate-solubilizing bacteria (PSB) are of central importance. NFB have the ability to convert inert N_2_ into ammonium and thereby protect nitrogen from being lost through volatilization and leaching [13]. PSB can convert insoluble phosphates into a bio-available form through solubilization and mineralization [14].

Soil C plays a crucial role in the application of bio-fertilizer, which is one of three soil components crucial for its physical and biochemical properties, and the degradation of organic matter is closely related to soil microbial activity [15,16]. Several studies have reported different findings regarding the effects of bio-fertilization on soil C content [17]. In turn, soil microbial populations and enzyme activities are related to organic C input (straw, compost, and manure), which could reduce the negative effects of the severe environment on microorganisms [18]. Many reports have highlighted the effects of microorganisms input on soil nutrient content, plant growth, and disease resistance, as well as the importance of soil C when applying microorganisms to the soil [9,16,19]. However, most approaches were conducted using a single bacteria strain, which may partially account for the recorded inconsistencies in the field [20,21]. Hence, less is known about the effects of co-inoculation with PSB and NFB on soil properties. The soil amending mechanism and interactions between NFB and PSB under C-deficiency remain to be determined.

The effects of bio-fertilizer evaluated in other areas are often limited by different factors, such as incubation time, inoculation types, limited C resources and survival of microbes [22]. On the other side, soil native microbes could influence the effects of bio-fertilizer on plant growth. Therefore, the characteristics of the typical soil in subtropical mountainous plantation areas, the time-effectiveness of inoculants, and the selection of the appropriate beneficial microorganism combination for fertilization should be investigated. The aims of this study were to determine the adaptive bacterial isolates or combinations of NFB and PSB, and to investigate the soil amendment mechanism and interaction between NFB and PSB under C-deficiency. Basically, a lab experiment was conducted to investigate, (1) whether these microorganisms could survive and multiplicate under limited C resources, (2) their efficiency in improving the main soil nutrient contents (N and P) in yellowish-brown clay soil under sterilized conditions. As supplementary, a pot experiment was conducted under non-sterilized soil conditions, to verify the effects of these strains accompanied by the native microbes, on plant growth and biomass accumulation. These results could interpret the mechanism of action and interaction between bacteria strains and soil with different incubation time under C-deficient conditions, as well as provide supports for the application of bio-bacterial fertilizer in such soils.

## 2. Materials and Methods

### 2.1. Soil Properties and Pretreatment

Natural soil was sampled from the topsoil (0–20 cm) at a *Cyclocarya Paliurus* plantation (a typical medicinal plant in subtropical regions in China) in July 2016, which was located in Baima Nanjing (31°35′ N, 119°10′ E), China. Samples were collected from five plots (1 × 1 m) in an “S” pattern in 4-year-old *C. Paliurus* plantation fields (about 120 × 40 m, at a planting density of 2 × 2 m) and were mixed thoroughly to form a composite sample. After removing the plant material, stones, and other debris, the collected soil was divided into two parts, one was sieved (2 mm) and kept at 4 ℃ prior to use in the lab experiment, the other one was used for pot experiment.

The above soil is the representative soil type in subtropical regions in China, which was classified as yellowish-brown clay soil with a heavy texture, pH of 6.5, bulk density of 1.6 g·cm^–3^, total C of 4.1 g·kg^–1^, total N of 0.79 g·kg^–1^, total P of 0.30 g·kg^–1^, total of K 0.10 g·kg^–1^, NH_4_^+^–N of 10.94 mg·kg^–1^, NO_3_^–^–N of 2.68 mg·kg^–1^, and available P of 1.03 mg·kg^–1^.

### 2.2. Microorganisms

In this study, we used four microorganisms, including phosphate solubilizing bacteria (PSB, viz., M: *Bacillus megaterium* and F: *Pseudomonas fluorescens*) and nitrogen-fixing bacteria (NFB, viz., C: *Azotobacter chroococcum* and B: *Azospirillum brasilence*). The above bacteria have been documented as having the ability to improve soil nutrients, such as N and P [23,24,25,26,27]. Prior to use, the inocula were prepared by incubating bacteria strains in a lysogeny-broth medium (LB medium, pH: 7.0, comprised of 10 g tryptone, 5 g yeast extract, and 10 g NaCl per liter). At the mid-exponential growth phase, the strains were diluted using sterile distilled water to a final concentration of 1 × 10^8^ colony forming units (CFU)·mL^–1^. None of these strains have shown antagonistic effects against one another [19,28,29].

### 2.3. Experimental Design

In the lab experiment, the soils were incubated with 12 additions (treatments) of the 4 bacteria, containing 4 treatments with a single bacteria addition (SBA), 7 treatments with a mixed bacteria addition (MBA), and 1 control with no bacteria addition (Table 1). Each treatment was replicated 4 times, and the bacteria were added to the soil, which was autoclaved enough times to eliminate other microbes. Thereafter, 300 g of sterilized soil supplemented with bacteria was placed into a cylindrical tissue-culture box (diameter (D) × height (H): 8.5 × 8.4 cm, breathable and waterproof), and the box was incubated in a bio-clean incubator at 28 °C under darkness conditions for 60 days. During incubation, the soil moisture was held at 60% of the water holding capacity with sterile water.

The pot experiment was conducted based on the lab experiment results, with three types of bacteria combination (PSB: M, MF; NFB: C, CB; PSB+NFB: MFCB). An important medicinal species (*C. Paliurus,* 2-year-old seedlings) native to China’s subtropical mountainous area, was grown in the same soil as we used in this study without sterilization. From April, four times of bio-fertilizations were conducted every 45 days according to bacterial growth results.

### 2.4. Sampling and Analytical Methods

**Figure 1:**
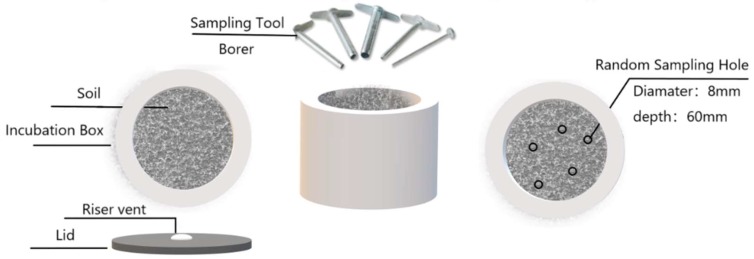
Abridged general view of soil sampling. Five random vertical sampling holes (diameter: 8 mm; depth: 60 mm) were implemented for lessening the disturbance of sampling to microbes.

The soils in the lab experiment were vertically sampled on the 5th, 10th, 15th, 20th, 30th, 45th, and 60th days of incubation (Figure 1) to estimate the bacterial quantity (BQ) using the plate count serial dilution method [30]. Similarly, soil samples from each box on the 0th, 30th, and 60th day of incubation were collected and stored at 4 °C for measurement of the soil properties. Total C (TC) and total N (TN) were evaluated using an elemental analyzer (vario MAX CN, Elementar, Hanau, Germany), where the concentration of inorganic N (IN, including NH_4_^+^–N and NO_3_^–^–N) was extracted with a 2 M KCl solution, and then measured by colorimetry on an AutoAnalyser III (SEAL Analytical, Berlin, Germany). Soil available P (SAP) was determined using the molybdenum-blue method [31]. Acid phosphatases (AcPase) activity was assessed using the method described by Tabatabai and Bremner [32]. Each experiment was conducted in three replicates for measurements of the BQ and soil properties.

For the measurement of plant growth in the pot experiment, the whole plants were sampled in late September to assess the biomass accumulations (including stem, root, and leaf). Seedling heights were measured by the difference of initial (April) and final height (late September).

### 2.5. Statistical Analysis

The Shapiro–Wilk test and Levene’s test were used for testing the normal distribution of the data and homogeneity of the variances, respectively. Mixed linear models were used to assess the effects of the inoculant, incubation time, and their interactions (as fixed effects), as well as the block as a random effect on the soil’s biochemical properties. Where there were significant effects (*p* < 0.05), the Duncan’s multiple range test was applied to determine the differences between the individual treatment means. Tamhane’s T_2_ was used to test for differences amongst treatments when variances of the tested data were not equal. Data are expressed as means ± standard deviation (SD). All statistical analyses were considered significantly at *p* < 0.05. The pairwise relationships of BQ and P-related indexes were elucidated using linear regression based on Spearman’s correlation analysis. All statistical analyses were performed using SPSS 19.0 (SPSS Inc., Chicago, IL, USA).

## 3. Results

### 3.1. Dynamic Growth of Bacteria in Incubation Soil

**Figure 2:**
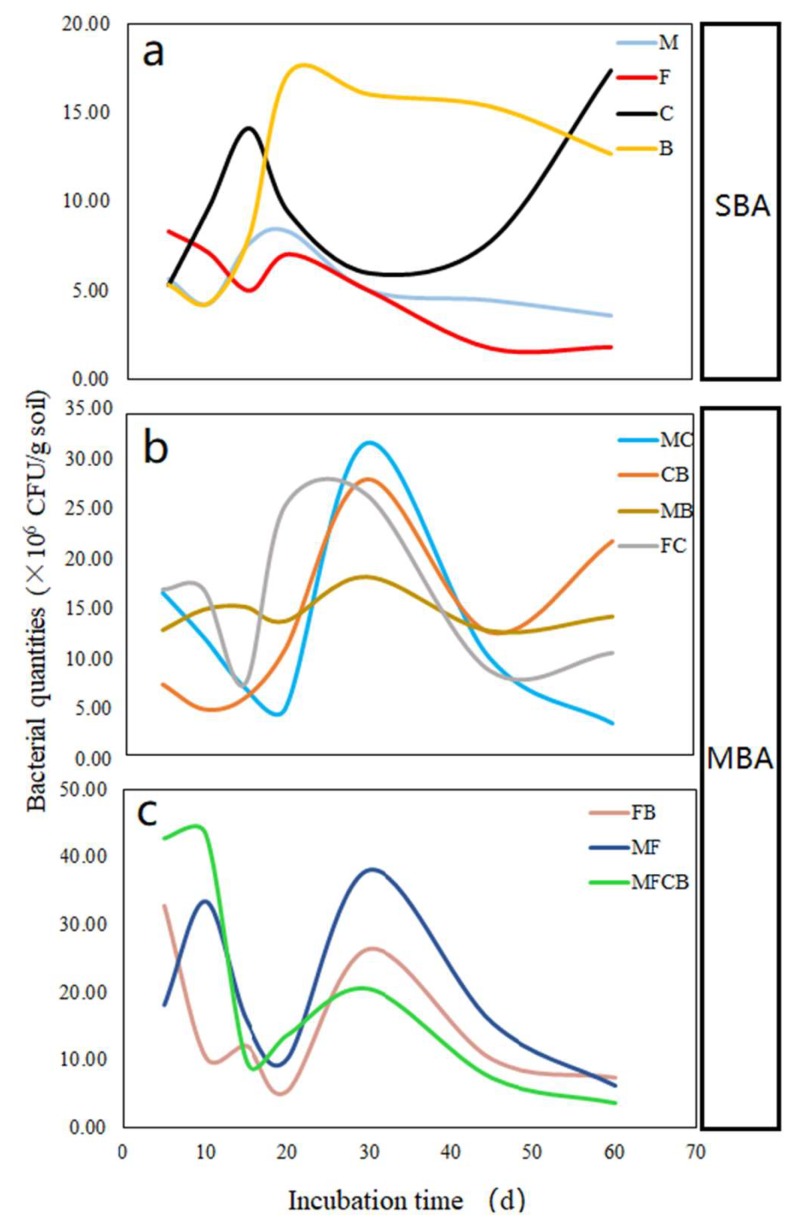
Three dynamic growth patterns of different inoculants during the 60-day incubation. (**a**) pattern 1, a single peak observed at 10–20 d by single bacterium addition. (**b**) pattern 2, a single peak observed at 30–40 d by mixed bacteria addition. (**c**) pattern 3, bimodal observed at different time by mixed bacteria additions.

Generally, the bacterial quantities (BQ) in all the treatments significantly increased with prolonged incubation, whereas the maxima were obviously different between the mixed bacteria addition (MBA) and the single bacteria addition (SBA). The maximum values of the BQ for MBA ranged from 18.3 × 10^6^ CFU·g^–1^ in MB to 43.3 × 10^6^ CFU·g^–1^ in MFCB, whereas for SBA, they ranged from 8.3 CFU·g^–1^ in M to 17.3 × 10^6^ CFU·g^–1^ in C (Table 2). Based on the dynamic changes in bacterial growth, three patterns were observed for the different additions (Figure 2). The peaking of the BQ for SBA occurred at different times from that in MBA; the quantity in SBA peaked at the 15–20th day and the peaks in most of the MBA (MC, CB, MB, FC) occurred at the 30th day, whereas some (FB and MF) presented bimodal peaks at the beginning and midterms of incubation (Figure 2).

Quantities of the two functional bacteria varied with incubation length. Quantities of the phosphate-solubilizing bacteria (M and F) appeared to decline in the last 30 days, while N_2_-fixing bacteria (C and B) increased (Table 2 and Figure 2a). On the 60th day, 17.3 × 10^6^ and 12.7 × 10^6^ CFU·g^–1^ in C and B, respectively, were significantly higher than the 1.9 × 10^6^ and 3.6 10^6^ CFU·g^–1^ in M and F, respectively (*p* < 0.05).

Overall, the single bacteria grew rapidly without competing pressure compared to other combinations, reaching their peak quickly and with a low maximum quantity. Conversely, the competition of mixed bacteria retarded the peaking time but increased the maximum.

### 3.2. Inoculants, Incubation Time, and Their Interactions on Soil Characteristics

Based on the statistical analysis results, the effects of the inoculants, incubation duration, and their interactions on soil TC, TN, IN, available P, and P-related enzyme activities are presented in Table 3. Over a 60-day incubation, we found that TC and TN showed significant responses to incubation time, whereas no significant effects of inoculant addition on TC and TN were detected (*p* = 0.07 and 0.06, Table 3). IN (NH_4_^+^–N + NO_3_^–^–N), available P, and P-related enzyme activities of the incubation soil were significantly affected by inoculant additions and incubation duration (*p* < 0.01). Interactions of the inoculants and incubation duration were significant for all measured parameters (*p* < 0.05, Table 3).

Given the significant effects of incubation time on these indexes, pairwise comparisons of the indexes between 30 days and 60 days were analyzed for all additions (Table 4). Impacts of incubation duration on SAP existed in each inoculant except CK. However, the effects of incubation duration on other soil parameters varied with different inoculants.

### 3.3. C and N Contents of the Incubation Soil

As shown in Table 5, the results show the significant effects of bacteria additions on TC and TN after a 30-day incubation (*p* < 0.05). Prominent increases in TN content were found in MF, FB, and FC at 30 days compared to the control (0.7 g·kg^–1^). However, variations in TC and TN contents among different bacteria additions were not significant at 60 days, but obvious reductions occurred in both SBA and MBA at 60 days compared to 30 days (Table 4; Table 5; *p* < 0.05). For instance, significant decreases in TC in treatments M, C, and MC were detected at 60 days compared to 30 days (decreased by 12.1%, 7.4%, and 16.1%, respectively), whereas obvious reductions of N in MF, MC, MB, FC, and FB were recorded (Table 5).

Differences in IN contents were observed after additions of various bacteria and two incubation durations (Table 4; Table 5). A significant increase in soil IN content was detected in the first 30 days after bacteria addition (Table 5, *p* < 0.05). However, in contrast to the 30 days, the IN contents of most treatments at 60 days were lowered but were still significantly higher than in the control (Table 4, *p* < 0.01). Statistically, no remarkable changes were detected under conditions of inoculation with NFB alone (except B at 30 days) compared to the control.

### 3.4. AcPase Activity and SAP Concentrations

**Figure 3:**
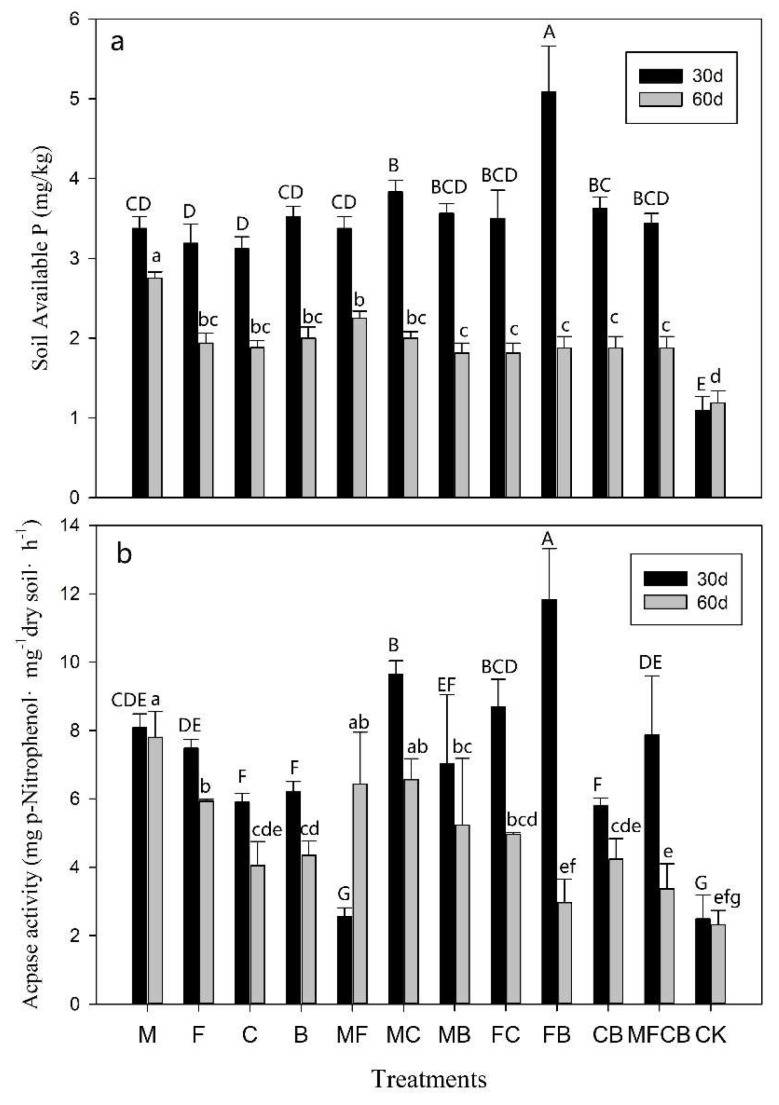
(**a**) Soil available phosphorus (SAP) contents and (**b**) AcPase activity of different treatments at the 30^th^ (black bar) and the 60th day (gray bar). Marked capitals/lowercases above the standard line mean the significant difference among different treatments at the 30th day/the 60th day (*p* < 0.05).

Soil available phosphorus (SAP) concentrations and AcPase activity in the soil after a 60-day incubation are presented in Figure 3. The SAP levels of all treatments were very low, ranging from about 1 mg·kg^–1^ in CK to 5 mg·kg^–1^ in FB at 30 days (Figure 3a). During the 60-day incubation, the SAP concentrations of all treatments increased at 30 days in contrast to CK, but significantly declined at 60 days (Table 4, *p* < 0.01). For example, the SAP in FB at 30 days was significantly higher than in other treatments, but then declined by about 63% at 60 days, which was in accord with the change in the corresponding AcPase activity (Figure 3b).

**Figure 4:**
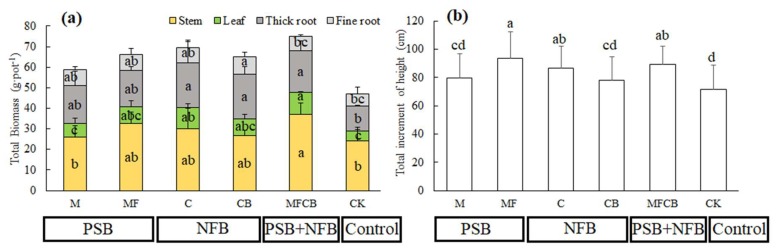
(**a**) Plant biomass (including stem, leaf, thick root, fine root) and (**b**) total increment of seedling height in different treatments with PSB (M and MF), NFB (C and CB) and PSB+NFB (MFCB).

Significant variations in AcPase activity in different treatments were detected (*p* < 0.05) and the impacts of incubation duration on AcPase activity in SBA were different from the effects in MBA (Table 4). For instance, AcPase activity in treatment M (belonging to SBA) showed no significant differences between 30 and 60 days, whereas AcPase activity in MFCB and FB (belonging to MBA) at 60 days showed lower values (Figure 4). AcPase activity and SAPs in single applications of NFB (C, B, CB) were lower than in most of the other additions, although co-inoculation with both PSB and NFB (MC, FC, FB, MFCB) significantly increased the concentrations of SAP and AcPase activity at 30 days (*p* < 0.05, Figure 3). However, this effect was minimal at 60 days. Compared to 30 days, the AcPase activity in FB at 60 days declined by 70%, which was accompanied by an obvious drop in the SAP concentration (Table 4, Figure 3; *p* < 0.01).

### 3.5. Seedling Height and Biomass Accumulation

As shown in Figure 4a, the total biomass accumulation of *C. Paliurus* was significantly increased after bacterial additions. Plant biomass assessment was divided into four components, including stem, leaf, thick root and fine root. Compared with seedlings grown in native soil (CK), significant increments were detected in each component after bacteria addition. However, no positive effect of PSB application (treatment M and MF) on plant biomass was found during the investigation. On the contrary, the application of NFB (C and CB) significantly increased biomass accumulation of leaf and root. It is noteworthy that the biomass of above ground (stem, leaf) and thick root in co-inoculation with PSB and NFB (treatment MFCB) obtained about 47.8g and 20 g per plant respectively, which were significantly higher than when these microorganisms were used alone.

Compared with CK, the total increments of seedling height were improved after bacterial additions (Figure 4b). Specifically, dual inoculation with PSB (MF) and co-inoculation with PSB and NFB (treatment MFCB) resulted in greater influences on seedling height than other treatments, including treatment only retained with native microbes.

## 4. Discussion

### 4.1. Changes of BQ Influenced by C Resources and Interactions of Bacteria

Shortages of available N and P in soils with poor C content are common in hilly and mountainous regions in China’s subtropical area where plant growth is limited. Bio-fertilization is a better choice compared to chemical fertilization for sustainably improving soil fertility [7,33]. Different from earlier studies [15,16], we incubated the inoculants in soil that collected from poor natural fields with low-level C. Soil biological properties, such as bacterial/fungal quantities and enzymes, are significantly correlated with soil C level [34]. As a result, BQ in most of the treatments performed similarly, with increases first then decreases during the 60-day incubation period (Figure 2). This indicates a coefficient restriction between limited C resources and the resilience of bacteria [35].

Here, three growth patterns of the inoculants were observed during the incubation, suggesting different responses of the BQ to inoculant isolates or in combination under C-deficient conditions (Figure 2). The BQ in some treatments increased again after their first peak, such as the co-inoculations (MF, FB, MFCB) in pattern 3 (Figure 2c). This was obviously different from previous publications in which only one peak was observed [20,21,36]. We speculated that the occurrence of the second growth of bacteria was mainly stimulated by co-inoculation with PSB and NFB, where synergistic effects activated under the circumstances of limited available C and N resources in the microcosms [19,37]. Similar studies reported that mixed microbial cultures allowed their components to interact with each other synergistically via physical or biochemical activities, thereby simultaneously improving viability [38,39]. In this experiment, synergistic mechanisms were found in the MF, FB, and MFCB, but BQ finally decreased under limited nutrients conditions. This result provides support when choosing the inoculant type (PSB+NFB) and frequency (30–45d) of fertilization when applying bio-fertilizer in such soils. Co-inoculation with PSB and NFB in soil results in more interactions of inoculants, such as the production of enzymes and organic acid, although more energy and inorganic nutrients would be consumed than when these organisms were used alone [19,40,41]. This was also supported by our study, where limited energy resources restrained the population growth for MBA at 30–40 days. Hence, the appropriate amount of C resource input during bio-fertilization is necessary when applying in such soil with low C level.

### 4.2. Additions of Bacteria Improved Soil Nutrients with Different Patterns

The responses of BQ to different inoculants under C-deficient conditions provided a better understanding of the relationship between the BQ, inoculant type, incubation duration, and available nutrients. Soil available nutrients, such as available N and P, are indispensable in regulating plant growth. However, soil available nutrients are often limited due to the changes in related enzyme and microorganism activities. During culture, the available nutrient contents in soil increased at an early stage (30 days) but declined at a later stage (60 days, Table 4; Table 5; Figure 3). This pattern was consistent with the changing tendency of BQ (Table 2). Many studies have shown that the populations of beneficial microbes in soil provided the foundations that positively affected soil characteristics [22,42,43]. Limited bacteria quantities in soil decreased available nutrients production, such as N and P, which may, in turn, restrict the population of microbes and affect the rates of the C decomposition process [44,45]. Related reports have revealed that soil available C and N affect the pivotal process of microbial growth, and N-assimilation that driven by soil microorganisms mostly occurs in the NH_4_^+^–N of inorganic N and alanine of organic N [46,47,48]. The microflora is positively correlated with soil C and available nutrients, and soil nutrients are conducive to increasing the abundance of soil microorganisms [49,50]. Thus, regular organic and bio-based fertilization of soils are favorable to the building of positive structures and functioning of the soil microbial community [3,51,52].

Few effects of a single application of NFB on the availability of N were detected during culture (Table 5). However, co-inoculations with NFB significantly increased soil available P concentrations and the related enzyme activity (Figure 3a). Two assumptions to explain these synergistic effects are presented here: (1) co-inoculants with NFB could synergistically stimulate population growth of microbes based on the above discussion and (2) NFB could directly promote the activity of P-related enzymes (AcPase). Liu et al. stated that certain species of NFB could increase P uptake under N addition, which is related to soil P-related enzyme activity [53,54]. AcPase activity is significantly affected by soil N, P conditions, and soil microorganism activities could result in an obvious change of AcPase activity. However, the AcPase activities of soil culture with NFB (C, B, CB) were obviously lower than the others (Figure 3b). This indicates the synergistic effect of specific NFB strains on SAP and related enzyme activity could be explained by stimulating growth and phosphate-solubilizing effects of PSB, rather than directly increasing the AcPase activity. This assumption could explain the result of the FB treatment, where the BQ decreased by about 72% at 60 days compared to 30 days, being accompanied by a drop in AcPase activity and SAP concentration.

**Figure 5:**
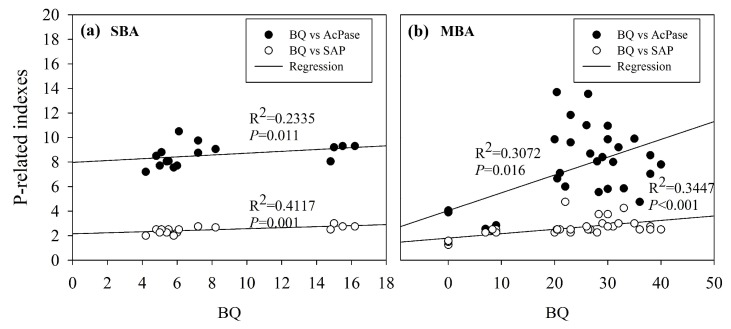
The relationships between (**a**) BQ vs P-related indexes in SBA at 30d, (**b**) BQ vs P-related indexes in MBA at 30 d on linear regression.

The relationships between BQ and P-related indexes in SBA and MBA at 30d based on linear regression are shown in Figure 5. The P-related indexes (SAP and AcPase) significantly increased in both SBA and MBA with increases in the bacterial quantities (*p* < 0.05, Figure 5), while MBA resulted in higher value. And the SAP concentrations were correlated with the AcPase activity (*R^2^* = 0.5423, *p* < 0.001). This suggests that changes in the SAP concentrations mainly resulted from changes in the BQ and following altered P-related enzyme activities under bio-fertilization.

### 4.3. Co-Inoculation with NFB and PSB Resulted in Higher Plant Biomass Accumulation

The pot experiment was used for evaluating the pragmatic effects of these bacteria by compared to treatment with only native microbes (CK), while the lab experiment was conducted for verifying whether these bacteria could survive and benefit the soil nutrients. Similar researches were found in many published literature papers, in which sterilized or oven-dried soil was used for testing the effects of beneficial microorganisms without disturbance of other microbes under controlled conditions, and non-sterilized soil was used for investigating the pragmatic effects on plants under natural conditions [55,56,57,58]. Plants accompanied by soil microorganisms in rhizosphere that could assist plants with nutrient acquisition [59]. Therefore, additions of bio-fertilizer improve the available nutrients supply for plant growth. Under natural conditions, compared with treatment with only native microbes, soil nutrient contents, and plant N and P uptake were significantly improved after bacteria addition, especially for treatment MFCB (co-inoculation with PSB and NFB). More importantly, the relative abundances of these bacteria were increasing at the first 30 days, but decreased after that (data not shown). This suggests these bacteria could survive and enlarge population during the initial competition with native microbes, but continuous bio-fertilization is necessary to help these microorganisms get advantage. In the present study, bacteria addition increased seedling height and biomass accumulation under unsterilized soil condition. More importantly, they increased the biomass of the whole plant, especially the biomass of the leaf, which is the most valuable organ for medicinal use. Based on these results, applications of bio-fertilizer, such as MFCB, in leaf-use plantations of *C. paliurus* could be a potential sustainable strategy for these plantations in the future.

To date, the interactive effects of co-inoculation with NFB and PSB on C-deficient soil conditions have been less studied. However, advanced mechanisms for interpreting the synergistic effects between NFB (*A. chroococcum*, *A. brasilence*) and PSB (*B. megaterium*, *P. fluorescens*) should be further investigated and evaluated to clarify the biochemical basis of these interactions. The survival and growth of strains vary with the chemical, physical, and biological differences between in vitro conditions and the field environment. A combination of NFB and PSB might cause competition for energy sources, such as root exudation and soil available nutrients. Hence, to obtain accurate conclusions about the effects of co-inoculation with NFB and PSB, further studies should be considered under different environmental media, and based on various research conditions [15,19].

## 5. Conclusions

Based on the results of the lab experiment and pot experiment, inoculation with beneficial bacteria had a positive effect on soil amendment and plant growth. Bacterial additions increased soil N and P availability, and co-inoculations with PSB and NFB enhanced the accumulation of the available P. However, decreases in soil nutrients were observed at 60 days compared to 30 days, which were induced by the decrease in bacterial quantities under C deficiency. These results highlight the interaction mechanism between strains and soil with the increase in the incubation duration under C-deficiency conditions. Besides, co-inoculations with PSB and NFB resulted in greater performance in plant growth promotion and nutrients uptake. In summary, aimed at amending the yellowish-brown clay soil with low levels of C, bacteria combinations (PSB+NFB) are recommended for practical application at intervals of 30–45 days. The lab experiment provided the basis for applying these microorganisms in natural environments, which helped us understand the possible interactions between PSB and NFB under C deficiency. The pot experiment results cross-validated that co-inoculation with PSB and NFB resulted in greater performance. This research gives the first interpretation of the mechanism of action and interaction between bacteria strains and soil under C deficiency, and contributes to the development of a biotechnological strategy, and sustainable agriculture, thereby minimizing the input of chemical fertilizers.

## Figures and Tables

**Table 1:** Soil with 12 additions with different bacteria combinations (mL).

Inoculants Type	Treatment Code	M: *Bacillus megaterium*	F: *Pseudomonas fluorescens*	C: *Azotobacter chroococcum*	B: *Azospirillum brasilence*
SBA	M	5	0	0	0
	F	0	5	0	0
	C	0	0	5	0
	B	0	0	0	5
MBA	MF	2.5	2.5	0	0
	MC	2.5	0	2.5	0
	MB	2.5	0	0	2.5
	FC	0	2.5	2.5	0
	FB	0	2.5	0	2.5
	CB	0	0	2.5	2.5
	MFCB	1.25	1.25	1.25	1.25
Control	Control	0	0	0	0

**Table 2:** Changing patterns of bacterial quantities (×10^6^ CFU·g^–1^ soil) for bacterium addition with different incubation duration (means ± standard deviation).

Treatment Code	Incubation Duration (d)							
	0	5	10	15	20	30	45	60
MFCB	0.167 ± 0	42.7 ± 5.3Aa	43.3 ± 4.7Aa	9.7 ± 1.5Dc	13.6 ± 3Cbc	20 ± 0.6Bd	7.3 ± 1DEbcde	3.7 ± 1Fefg
MF	0.167 ± 0	18C ± 0.3c	33.3 ± 1.9Bb	15.67 ± 1.3Da	10 ± 1.2Ecd	38 ± 1.2Aa	15.33 ± D1a	6.1 ± 1Fef
FC	0.167 ± 0	17 ± 6Bc	17 ± 5.5Bc	7.2 ± 1DEde	25.6 ± 3.8Aa	26.6 ± 3.2Ac	8.7 ± 2.8CDbc	10.5 ± 2Ccd
MB	0.167 ± 0	12.9 ± 2BCc	15 ± 1.6ABc	15.3 ± 2ABa	13.8 ± 1Bc	18.3 ± 2Af	12.8 ± 2.7BCab	14.3 ± 0.3Bc
FB	0.167 ± 0	33 ± 4.3Ab	10.3 ± 4.1Ccde	12 ± 2Cb	5.3 ± 1DEefg	26.3 ± 3.5Bc	10 ± 3.2Cabc	7.3 ± 2Dde
CB	0.167 ± 0	7.3 ± 2Dd	4.8 ± 2.3DEFde	5.9 ± 1.8DEde	11 ± 1Ccd	28.3 ± 5.7Ac	12.7 ± 5Cab	22 ± 5.3Ba
MC	0.167 ± 0	16.7 ± 5Bc	12.1 ± 0.2Ccd	7 ± 1DEde	4.8 ± 1.8EFfg	32 ± 2.6Ab	10 ± 3.5CDabc	3.3 ± 1FGefg
B	0.167 ± 0	5.3 ± 0.6Ed	4.3 ± 2.2EFe	8.0 ± 1Dde	17.2 ± 3.5Ab	16 ± 0.8ABe	15.3 ± 4ABCa	12.7 ± 3Cc
C	0.167 ± 0	5.3 ± 3.2DEFd	9.7 ± 1.5Ccde	14.0 ± 0.5Bc	9.3 ± 3Cde	6.0 ± 1DEf	7.7 ± 1CDbcd	17.3 ± 2Ab
F	0.167 ± 0	8.3 ± 1.5Ad	7.1 ± 2.5ABde	5.0 ± 0.4BCe	7.1 ± 3ABdefg	5.0 ± 0.8BCf	1.8 ± 0.8De	1.9 ± 0.7Dg
M	0.167 ± 0	5.6 ± 1.5BCd	4.2 ± 1.6CDEe	7.7 ± 1.5ABcd	8.3 ± 3Adef	5.0 ± 0.6CDf	4.5 ± 0CDEcde	3.6 ± 1DEefg

M, F, C, B: single inoculation with M (*Bacillus megaterium*) or F: (*Pseudomonas fluorescens*) or C (*Azotobacter chroococcum*) or B (*Azospirillum brasilence*). MC, CB, FB, MB, FC, MF: dual inoculation with M and C, C and B, F and B, M and B, F and C, M and F. MFCB: mixed inoculation with four strains. Different capital letters denote significant differences among incubation durations at *p* < 0.05, different lowercase letters denote significant differences among treatments at *p* < 0.05 on the same incubation duration.

**Table 3:** The linear mixed model for the effects of inoculants, incubation time, and their interactions on soil characteristics.

Variables	Inoculants		Incubation Duration		Inoculants × Incubation Duration	
	F-test	*Sig.*	F-test	*Sig.*	F-test	*Sig.*
TC	1.82	nd	54.48	**	2.03	*
TN	1.86	nd	43.11	**	2.93	**
NH_4_^+^-N	10.90	**	24.57	**	3.11	**
NO_3_^-^-N	20.27	**	48.50	**	5.87	**
SAP	26.58	**	2162.43	**	21.40	**
AcPase	24.42	**	67.18	**	21.34	**
IN	19.18	**	75.34	**	7.65	**

TC: total carbon; TN: total nitrogen; SAP: soil available phosphorus; IN: inorganic nitrogen; *Sig*: significance, * indicates *p* values < 0.05, ** indicates *p* values < 0.01, nd indicates significance not dectected.

**Table 4:** Pairwise comparisons’ results of soil indexes between 30d and 60d incubation for all additions.

Inoculants		TC	TN	NH_4_^+^-N	NO_3_^-^-N	SAP	AcPase	IN
		30d-60d	30d-60d	30d-60d	30d-60d	30d-60d	30d-60d	30d-60d
SBA	M	**	**	**		**	nd	**
	F	nd	*	*	*	**	*	**
	C	*	nd	nd	nd	**	nd	nd
	B	nd	nd	**	nd	**	nd	**
MBA	MF	**	**	**	*	**	**	**
	MC	**	*	nd	**	**	**	**
	MB	*	*	nd	**	**	*	nd
	FC	*	**	nd	**	**	**	nd
	FB	*	**	*	**	**	**	**
	CB	nd	nd	*	nd	**	*	**
	MFCB	nd	nd	*	nd	**	**	**
Control	CK	nd	nd	nd	nd	nd	nd	nd

**Table 5:** Incubation soil TC, TN and IN contents after beneficial bacteria addition.

Inoculants		TC (g·kg^–1^)		TN (g·kg^–1^)		IN (mg·kg^–1^)	
		30 d	60 d	30 d	60 d	30 d	60 d
SBA	M	4.6 ± 0.2a	4.0 ± 0.3a *	0.9 ± 0.1a	0.7 ± 0.1a *	34.8 ± 1.6b	19.6 ± 2.4cd *
	F	4.1 ± 0.2a-d	3.9 ± 0.1a	0.9 ± 0.1ab	0.7 ± 0.1a *	34.3 ± 2.5bc	23.9 ± 6.6abc *
	C	4.3 ± 0.2a-d	4.0 ± 0.2a *	0.7 ± 0.1c	0.7 ± 0.1a	10.7 ± 2.8f	9.7 ± 3.5f
	B	4.1 ± 0.1cd	3.9 ± 0.1a	0.8 ± 0.1bc	0.7 ± 0.1a	30.4 ± 8.5cd	8.7 ± 1.2f *
MBA	MF	4.5 ± 0.3ab	4.0 ± 0.6a *	0.9 ± 0.0a	0.6 ± 0.1a *	43.1 ± 4.8a	28.6 ± 3.6ab *
	MC	4.5 ± 0.4a	3.8 ± 0.1a *	0.8 ± 0.1abc	0.7 ± 0.1a *	23.7 ± 7.4de	17.5 ± 4.2cde *
	MB	4.4 ± 0.1abc	3.9 ± 0.1a *	0.8 ± 0.1abc	0.7 ± 0.1a *	25.9 ± 5.5d	22.3 ± 2.2bc
	FC	4.2 ± 0.2a-d	3.8 ± 0.1a *	0.9 ± 0.1ab	0.7 ± 0.1a *	22.4 ± 3.4de	18.4 ± 4.9cde
	FB	4.2 ± 0.1bcd	3.8 ± 0.1a *	0.9 ± 0.1ab	0.7 ± 0.1a *	27.7 ± 1.3cd	18.9 ± 2.5cd *
	CB	4.2 ± 0.1cd	4.2 ± 0.1a	0.7 ± 0.1c	0.7 ± 0.1a	21.2 ± 4.7bc	12.2 ± 0.9def *
	MFCB	4.3 ± 0.1a-d	4.1 ± 0.1a	0.8 ± 0.1abc	0.7 ± 0.1a	20.2 ± 0.2e	30.8 ± 12.8a *
CK	CK	4.0 ± 0.1d	4.0 ± 0.2a	0.7 ± 0.01c	0.7 ± 0.0a	10.9 ± 1.4f	10.8 ± 0.1ef

Different lowercase letters denote significant differences among treatments at *p* < 0.05 on the same sampling date. * means significant differences between 30d and 60d.
